# Inteins in Science: Evolution to Application

**DOI:** 10.3390/microorganisms8122004

**Published:** 2020-12-16

**Authors:** Ananya Nanda, Sourya Subhra Nasker, Ashwaria Mehra, Sunita Panda, Sasmita Nayak

**Affiliations:** School of Biotechnology, Kalinga Institute of Industrial Technology, Bhubaneswar 754021, Odisha, India; ananya1992@gmail.com (A.N.); souryasubhra96@gmail.com (S.S.N.); ashwariamehra1996@gmail.com (A.M.); sunitapanda686@gmail.com (S.P.)

**Keywords:** inteins, splicing, evolution, application, protein engineering

## Abstract

Inteins are mobile genetic elements that apply standard enzymatic strategies to excise themselves post-translationally from the precursor protein via protein splicing. Since their discovery in the 1990s, recent advances in intein technology allow for them to be implemented as a modern biotechnological contrivance. Radical improvement in the structure and catalytic framework of *cis*- and *trans*-splicing inteins devised the development of engineered inteins that contribute to various efficient downstream techniques. Previous literature indicates that implementation of intein-mediated splicing has been extended to in vivo systems. Besides, the homing endonuclease domain also acts as a versatile biotechnological tool involving genetic manipulation and control of monogenic diseases. This review orients the understanding of inteins by sequentially studying the distribution and evolution pattern of intein, thereby highlighting a role in genetic mobility. Further, we include an in-depth summary of specific applications branching from protein purification using self-cleaving tags to protein modification, post-translational processing and labelling, followed by the development of intein-based biosensors. These engineered inteins offer a disruptive approach towards research avenues like biomaterial construction, metabolic engineering and synthetic biology. Therefore, this linear perspective allows for a more comprehensive understanding of intein function and its diverse applications.

## 1. Introduction

Splicing mechanism can be broadly categorized as RNA splicing and protein splicing, two mechanisms responsible for the flow of information from a gene to its protein product to yield a functional protein whose sequence is strictly noncolinear with the gene. While group I introns self-splice at a precursor RNA level, intein splicing involves the removal of an intervening sequence at a precursor polypeptide level [1]. Initially, this intervening polypeptide sequence was termed as spacer or protein introns, currently termed as inteins (INTervening protEINS) [2]. In a radical post-translational event, inteins excise themselves precisely from a larger precursor protein by sequential cleavage of peptide bonds and concomitant ligation by peptide bond formation between the flanking amino-terminal (N-) and carboxy-terminal (C-) residues termed as exteins, resulting in the formation of an active protein product [3,4]. The intein-mediated splicing mechanism lacks the use of any exogenous cofactors or high-energy molecules [3,5]. The embodiment of intein-mediated protein splicing in the “central dogma” of molecular biology puts in an additional level of complexity to the mechanism of gene expression [6].

Most of the inteins are interrupted by a homing endonuclease domain (HED) [7,8,9]. However, the HED can be removed from within the intein, without entirely compromising the splicing activity [10]. Thus the presence of HEDs increases the allele frequency at a rate higher than that of Mendelian rates [2,11]. Homing endonucleases encoded within an intein contains the prefix “PI-” in terms of intein nomenclature [12,13]. Conventionally, intein nomenclature comprises abbreviated names of both genus and species followed by the name of the protein; an intein residing in the GyrA protein of *Mycobacterium xenopi* is designated as *Mxe* GyrA, for instance [13,14]. *Mxe* GyrA, coincidentally, is also the smallest known naturally occurring intein [15].

**Figure 1:**
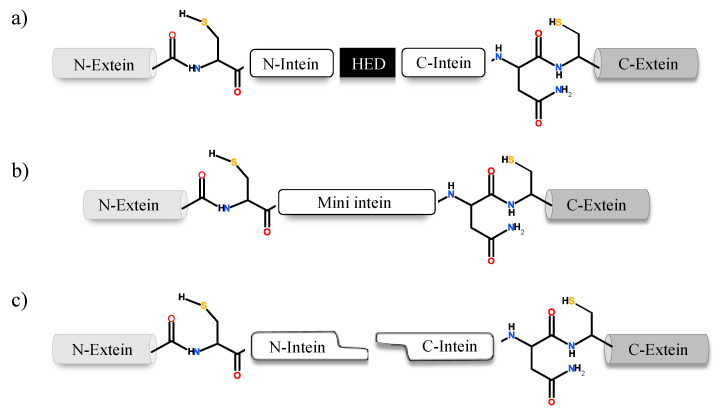
Intein configurations. Schematic representation of various types of intein: (**a**) full length intein with Homing endonuclease domain (HED), (**b**) mini-intein and (**c**) split intein.

Inteins naturally exist in three different configurations (Figure 1): (1) full-length inteins, where a sequence-specific homing endonuclease domain is embedded in between the splicing (catalytic) domains; (2) mini-inteins, lacking the homing endonuclease domain and containing a contiguous protein splicing domain; and (3) split inteins, transcribed and translated as two separate polypeptides each joined with an extein [4,8,16,17]. The study of intein distribution, dissemination and their potential biological functions are particularly fascinating in the field of translational research. Inteins distribution is sporadic in the genomes of organisms spanning from archaea, bacteria and eukaryotes to several viral genomes [18,19,20]. The reason for such anomalous distribution has spurred the proposal for numerous evolutionary scenarios, including the role of inteins in genetic mobility and as a selfish DNA [19,20,21]. Still, the question remains as to why inteins persisted for millions of years? Do they perform a beneficial role in the host or are they just a selfish gene? This phenomenon is puzzling and needs to be explored further.

The potential to exploit inteins for a practical purpose has led to the development of a diverse array of applications in modern biotechnology. Inteins can be engineered to undergo conditional protein splicing (CPS) which requires environmental or molecular triggers like light, changes in pH or temperature, change in redox state or addition of small molecules [22,23,24,25,26,27]. The bias nature of inteins toward plant and human pathogens makes it an attractive tool for novel drug development [28,29,30]. Development of engineered inteins or synthetic intein systems has encouraged efficient protein purification, ligation and cyclization strategies [9,31,32]. Recent advances in intein research have extended these in vitro application to whole organisms [9]. Such developing applications suggest that inteins are becoming a mature and critical biological tool, capable of widening the aperture to new avenues of scientific research, including enhanced transgenic plants and novel therapeutic strategies [9,28,33].

## 2. Intein Distribution and Evolution

**Figure 2:**
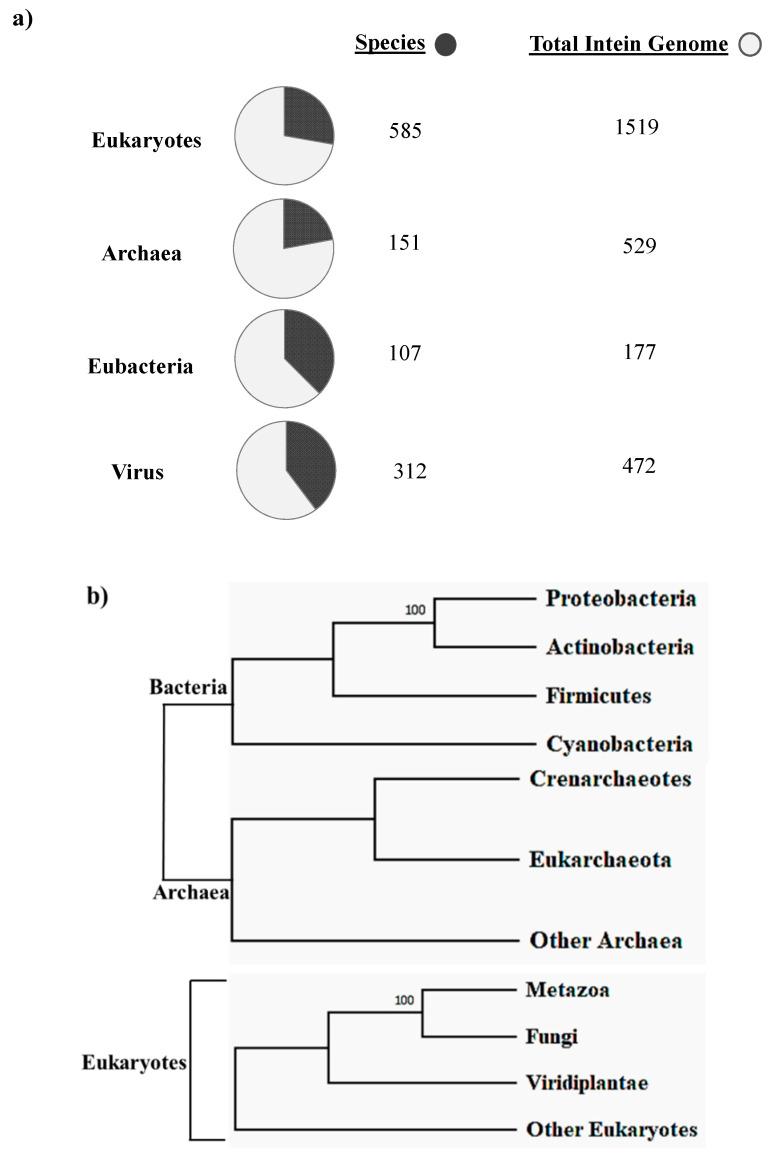
Sporadic distribution of inteins. (**a**) Summary of intein distribution with the total number of intein-containing genome from respective species indicated. The intein distribution data were extracted from the NCBI Gene data base. (**b**) Schematic representation of the tree of life showing four phyla for bacteria, three phyla for archaea and three kingdoms of eukarya (Metazoa, Fungi and Viridiplantae). All other eukaryotes are shown with the basal branch. Intein-containing gene sequences were obtained from NCBI and analyzed by MEGA-X software. The phylogenetic tree was constructed using the neighbor-joining method.

The first intein sequence was discovered 32 years ago in the *Saccharomyces cerevisiae VMA1* gene that encodes for an alpha subunit of vacuolar H^+^ ATPase [34,35]. The translational product of the gene was calculated to be 118.6 KDa but experimentally estimated as 67 KDa. The deduced amino acid sequence shows similarity to other ATPase at N- and C-terminal regions, but the central region was not determined [34]. Experimental analysis by Kane et al. revealed the presence of two separate proteins of molecular weights 69 and 50 kDa [35]. Since then, further examples of inteins were found in all three domains of life—in archaea, the DNA polymerase of the extremely thermophilic archaebacteria *Thermococcus litoralis* [36], in bacteria, the RecA proteins of *M. tuberculosis* [37,38] and *M. leprae* [39] and in eukarya, the 69 kDa subunit of vacuolar ATPase of the yeast *Candida tropicalis* [40]. This highlights a wider distribution of inteins across all three domains of life (Figure 2b), suggesting an ancient origin that predates the separation of prokaryotes and eukaryotes [18,20,41]. We dug into the NCBI Gene data base (www.ncbi.nlm.nih.gov/gene) to scan the distribution of intein in all the three domains of life, where out of 2709 intein-containing genomes, 56% of total intein-containing genome is found in eukaryotes, 19.8% in archaea and 6.64% in eubacteria. We also performed an assessment for intein distribution in viruses and observed 17.4% of total intein-containing genome is present in viruses (Figure 2a).

Novikova et al. performed a large-scale survey in order to analyze intein presence across bacteria and archaea. The survey revealed that half of the total archaeal genomes analyzed had at least one intein; in contrast, only a quarter of bacteria were found to be intein positive among the total bacterial genome studied [18]. A recent study conducted by Kelly et al. sheds light on intein distribution across bacteria and their phages. This analysis provides the first clear evidence of mycobacteriophages as major facilitators of intein dissemination across all of mycobacteria. The study found that 19.1% of mycobacteriophages contain inteins residing mostly in nucleic acid binding proteins, enriched in specific clusters [42]. Regardless of the exiguous presence of inteins in eukaryotes as reported by bioinformatics analysis, there is, however, intein presence in the fungal nuclear genome, algal chloroplast genome and within few eukaryotic viruses [41]. There is, however, a preponderance of inteins observed in fungi, mostly in Ascomycota representing some noteworthy pathogenic fungi, such as *Candida* sp. [16] and *Aspergillus* sp. [43]. Among others, inteins found in Basidiomycota include human pathogens, such as *Cryptococcus neoformans* and *C. gattii* [43,44,45] and plant pathogens *Tilletia indica* and *T. walkeri* [46]. The chloroplast DNA of diverse algae and seaweeds contains a staggering number of inteins in the Rhodophyta, Chlorophyta, Cryptophyta, Ochrophyta and Heterokonta phylums [41]. Amidst known eukaryotic viruses, there are hundreds of intein across four families, namely, Iridoviridae, Marseilleviridae, Phycodnaviridae and Mimiviridae [47]. Aforementioned fungal pathogens have intein presence commonly in Prp8 (pre-mRNA processing factor 8), VMA1 (vacuolar ATPase, subunit A), DnaB (DNA replication helicase DnaB-like), DdRP (RNA polymerase subunit beta RpoB), DdDP (DNA polymerases) and RIR (Ribonucleoside-diphosphate reductases) [41].

The primary indication of intein origin lies in its two-domain structure, suggesting that a mobile intein is a result of a fusion between two proteins, most likely, a self-splicing intein and an endonuclease protein. Sequence and mutational studies reported that the endonuclease activity is concentrated in the central portion of the intein, whereas the splicing activity is located in the two terminal regions [48,49]. However, it remains unclear whether an intein came first or the autocatalytic self-splicing domain in regulatory proteins [2]. Xiang-Qin Liu stated that a self-splicing mini-intein shows a correspondence between its structural and functional composition [21]. A mini-intein structurally consists of two subdomains along with a loop exchange between the same. Functionally, the splicing pathway consists of two peptide cleavages and a coupling between the two cleavages. This is not rather coincidental but suggests a structure–function relationship of the mini-intein. Liu further hypothesized that a fusion between two coding sequences gives rise to a duplication event in the domain responsible for the self-cleaving activity [21]. This fusion protein retains its biological property to perform self-cleavage independently. It may be that the homing endonucleases invade such an element later on. This idea is supported by the reason that endonucleases, being mobile in the genome, although remove themselves from the gene product but would account for a preferable integration site in these locations since the function encoded by the surrounding genetic elements would not be disrupted [1]. It is reasonable to think that naturally occurring mini-inteins most likely evolved from bifunctional mobile inteins by losing their endonuclease domain, because once an intein enters a host protein, there is no considerable selection pressure to maintain endonuclease activity, but a strong selection pressure for maintaining the splicing activity [21]. A split-intein may evolve from a mini-intein by initiating a break in the intein’s coding region [33]. The discovery of naturally occurring split-intein in a cyanobacterial DNA polymerase (DnaE) supports the idea. The N- and C-exteins of DnaE are linked to their respective intein fragment. It is, however, encoded by two separate genes located on different parts of the genome [50].

Interestingly, inteins are biased towards invading regulatory proteins that are responsible for DNA metabolisms (polymerases, topoisomerases, helicases, ribonucleotide reductases) and essential housekeeping genes, including essential proteases, metabolic enzymes, RNA processing proteins and energy supplying vital proteins. Their insertion site coincides with the conserved domains, responsible for host protein function like catalytic or ligand binding sites, enzyme active site, DNA binding sites etc. [2,18]. Insertion at these critical sites ensures the survivability of inteins, making them less prone to deletions. This site-specific behaviour of intein insertion may be due to the functionality of its homing endonuclease domain [10]. The amount of information conceived regarding the genome organization and expression of inteins in the last two decades has led to the understanding as to how mobile genetic elements are not solely parasitic sequences, but also have a dynamic role in the evolution of species.

## 3. Intein Structure

**Figure 3:**
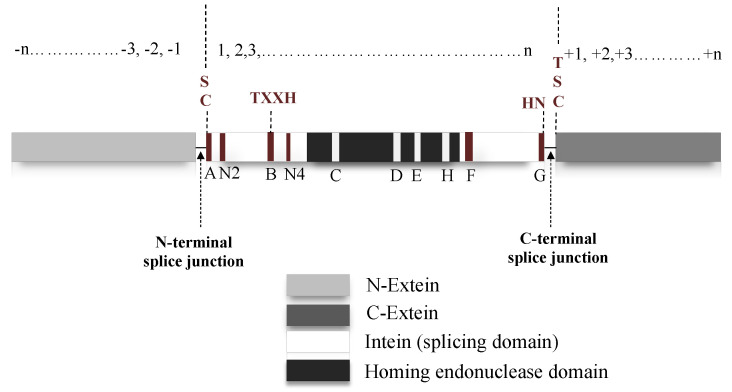
Schematic diagram displaying different structural domains of precursor protein. Intein insertion divides the host protein into N-extein (light grey) and C-extein (dark grey). Intein has two structural domains: splicing domain (white) and homing endonuclease domain (HED) (black). Residues on the intein and exteins are numbered as follows; Intein residues (1, 2, … n), N-extein residues (−1,−2, … −n) and C-extein residues (+1, +2, … +n). Centrally located endonuclease domain carries C, D, E and H conserved Blocks (motifs). N-terminal intein contains A, N2, B, and N4 Blocks (maroon Blocks), while F and G-Blocks (maroon Blocks), are seen in the C-terminal intein. Conserved residues within these regions either directly participate or assist in the cleavage and splicing reactions.

Natural intein structure is directly congruous to their functional role. If classified based on domain structure, inteins can be either full-length, mini-intein or split intein. Full-length intein bearing systems are expressed in a single polypeptide chain, which includes an intein domain responsible for splicing activity and a HED with a role in DNA invasion into the precursor protein-coding gene (Figure 3) [51,52,53]. Mini inteins, however, are ideally N- and C-terminal splicing domains joined together without the HED in between [10,16]. On the contrary, split-inteins contain two short fragments: N-terminal intein (I_N_) linked to N-extein and the C-terminal intein (I_C_) with corresponding C-extein. The two fragments reassemble into a complete intein structure that functions similar to a full-length intein system [50,54,55]. When inteins express themselves as a contiguous system, such as in a full-length intein, it is known as *cis*-splicing [20,56]. The split-intein system, expressed as two separate genes follow a *trans*-splicing system instead. Upon translation, the two separate polypeptides associate in a zipper-like network prior to excision from precursor [8,57,58]. Despite harbouring structural differences, both *cis*- and *trans*-splicing inteins follow a similar pathway to carry out splicing post precursor reestablishment [56]. Mini-inteins which typically have a continuous splicing domain lacking the HED, also follow a *cis*-splicing pathway [41,59,60]. An intein fragment consists of an N-terminal and a C-terminal end; upstream to the N-terminal intein the polypeptide sequence is termed as N-extein and downstream to the C-terminal intein is the C-extein. Residues on the intein are numbered sequentially from 1 to n (Figure 3). Extein residues are numbered in compliance with the intein sequence, the N-extein residues are numbered as −1 to –n from N-extein–intein junction. Residues on the C-extein are numbered as +1 to +n from the C-intein–extein junction [14]. The intein protein family belongs to the Hedgehog INTein (HINT) superfamily, named after the characteristic fold identified in Hedgehog and intein protein domains [61].

There is a homology between the splicing domain of intein and the HINT family protein-processing domain [61]. The HINT domain families can be classified into four major types: Hog-Hint, intein and two types of bacterial intein-like (BIL) domains. HINT superfamilies share similar biochemical activities, structural fold and common sequence features [62]. Inteins in their native form assume a horseshoe-shaped structure with an active site (catalytic fold), consisting of the splicing domain and conserved active-site residues. The active site brings the N- and C-terminal splice junctions within catalytic range, thereby commencing cleavage and subsequent splicing reaction [63].

The splicing domain of intein is broadly divided into two subdomains: N- and C-terminal splicing domains. These regions consist of several conserved motifs containing conserved residues, which facilitate the initiation and completion of splicing activity (Figure 3). N-terminal intein contains A, N2, B and N4 structural motifs or Blocks, while F and G-Blocks are seen in the C-terminal intein [8,13,52,53]. A-Block typically has Cys/Ser or Thr as conserved residues. B-Block usually contains His and Thr residues and F-Block usually has Asp and His. Additionally, G-Block bears two conserved residues; a penultimate His and a terminal Asn. These are the critical residues that directly or indirectly assist in the splicing reactions. C, D, E and H-Blocks are dedicated for the homing endonuclease domain [13,53,64,65].

HINT domain appears to be the key player in the intein-mediated splicing mechanism since it was shown to fold into a horseshoe-like structure similar to inteins. Intein is mostly limited to archaea, bacteria and unicellular eukaryotes, but Hog-Hint domains are spread across multicellular animals [20]. BIL domains have an overlapping phylogenetic distribution with inteins, spread among different bacterial proteins [62]. The following details illuminate the evolutionary standpoint of the Hog-Hint and BIL domains.

Hedgehog signaling developmental proteins in animals have three distinct protein domains [62]. The N-terminal or Hedge domain, which is a developmental signal cleaved from the precursor protein with subsequent covalent attachment of lipid on both ends and then secreted by the cell. The C-terminal or Hog domain is further subdivided into a HINT domain and a sterol-recognition region (SSR). The peptide bond shift is orchestrated by the HINT domain, leading to its attachment to Hedge domain through a thioester bond. The SSR attacks the thioester bond resulting in the cleavage of the Hedge domain followed by ester bond formation between the cholesterol molecule and the resulting C-terminus [66]. Crystal studies conducted by Hall et al. reported that Hog–Hint domains share the same structural and sequence motifs as their intein counterparts [61]. Few additional motif presence, such as an active site with Asp or His residue responsible for cholesterol activation, help distinguish the Hog–Hint domain among other HINT domains in the family [67].

A past study by Amitai and team members identified new bacterial intein-like (BIL) domains termed as A- and B-type BIL [68]. In terms of sequence motifs, the members of each BIL type show more similarity between one another; however, these are as different from each other as compared to other types of HINT domains. Unlike inteins, A- and B-type BIL domains are present in the hypervariable regions of the nonconserved bacterial proteins. The autocleavage property of BIL at N- or C-terminal is very similar to that of an intein. A-type BIL domains contain highly conserved His-Asn residues at their C-terminal ends, but their positioning is diverse. A-type BIL domains although can cleave their C-terminus with the help of Asn, but cannot ligate the flanking sequences [69]. However, B-type BIL domains share all motifs of intein splicing except for the C-terminal motif. B-type BIL can cleave their N-terminal domain, resembling the intein cleavage reaction, but the C-terminal cleavage follows an atypical reaction unlike in the case of an intein [68,69]. Aforementioned features suggest a difference in the biological role of BIL domains to that of an intein, where the former domain contributes mostly towards post-translation protein variability together with genetic rearrangements in microevolution.

The HED is located in between the N- and C-terminal inteins and is involved in the horizontal transfer of inteins to inteinless alleles termed as “intein homing” [12]. The coalition between the splicing element and endonuclease gives rise to a molecular parasite like structure, which has to be identified as a distinct evolutionary unit whose destiny, although entwined, remains separate from the host protein [70]. The HED can recognize and cleave up to a span of 12–40 bp of DNA showing a significant difference in recognition properties as compared to a restriction enzyme, which cleaves much shorter span of DNA in the 3–8 bp range [71]. There are four distinct families of homing endonuclease genes based on their conserved amino acids sequences: H-N-H, His-Cys box, the GIY-YIG and LAGLIDAG(DOD) type of endonucleases; LAGLIDADG endonuclease are the most commonly found sequence in various inteins [12,48,72,73]. HED initiates intein homing by cleaving the intein-less alleles inducing a double-strand break repair by homologous recombination, ensuring stable intein incorporation and long-term stability to persist in the population [7,72]. However, some past research shows evidence that the removal of HED can lead to inactivation of *Methanococcus jannaschii* TFIIB *cis*-intein indicating its mutualism in the protein splicing process [7,10].

**Figure 4:**
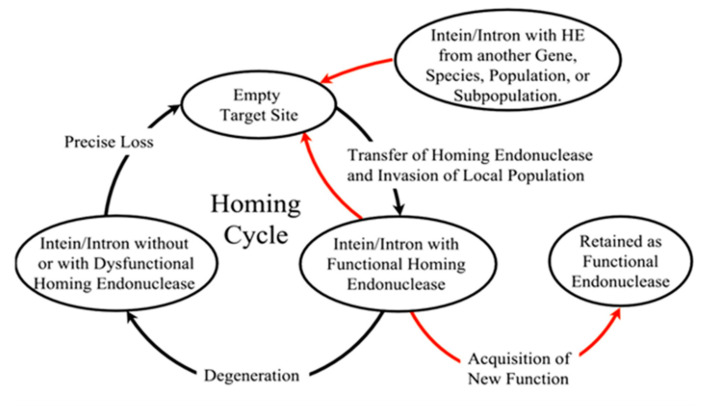
Homing cycle of a parasitic genetic element. Recent findings suggest that due to complex population structure the cycle might not operate in synchrony in different subpopulations. The red arrows indicate the trajectory of the functioning HE and the black arrows indicate the fate of the host gene. The precise loss can occur through recombination with an intein- or intron-free allele, or, in the case of introns, through recombination with a reverse transcript of the spliced mRNA [51].

The homing cycle or the life cycle of inteins with homing endonucleases was first formulated by Goddard and Burt [74], as they stumbled upon introns jumping frequently within different yeast species. They concluded that the cycle began with (a) an invasion of endonuclease-containing genetic element into an empty site by homing, lateral transfer or attaching of the invaded allele in the population; directly followed by (b) either loss or degradation of the endonuclease ORF, and then by (c) precise deletion of the parasitic genetic element and finally, step (a) all over again. However, Gorgaten et al. revised the cycle reinvasion model for endonuclease maintenance (Figure 4) and added that the empty sites can be occupied by a parasitic element from another gene or a subpopulation [51]. Thus, this homing cycle explains how a functional endonuclease maintains itself within a population for a long period. It has been questioned for years whether the long-term survival of the HED in a population adds a cost to the fitness of an organism.

The relative fitness of an organism directly correlates to the presence or absence of homing endonuclease and intein. Butler et al. had a suggestion that inteins may decrease the fitness of a host organism [43]. Although according to the rock–paper–scissor theory proposed by Brazel et al., while considering the fitness cost of three alleles: X with an empty target site, Y with a dysfunctional homing endonuclease and Z with a functional homing endonuclease. Carriers of X-allele will be more fit, followed by carriers of Y-allele. Between X- and Z-allele, however, due to the functional presence of homing endonuclease, there is a super Mendelian inheritance resulting in carriers of Z-alleles being more fit [70]. Adit Naor et al. further demonstrated experimentally to quantify the fitness cost associated with an intein. The research team used *pol*B-c intein in *Haloferax valcanii* and found a fitness cost of over 7%. In a direct completion assay performed on intein-positive and intein-negative strains, it was seen that the intein-negative strains outcompeted the intein-positive strains due to a faster growth rate [75]. A study conducted by Mills and coworkers in 2020 confirmed that mutations of the conserved residue in HED reduces splicing activity and depreciates growth in *H. volcanii* [76]. There is still an ongoing debate as to whether the molecular parasite’s selfishness is the driving force for its survival at a cost of the individual since it is highly likely that the molecular parasite is benefited but not the organism that ensures its survival. Researchers all across the globe ran hypotheses and experimental data to come up with a plausible theory.

## 4. Intein Splicing

Inteins implement classical enzymatic strategies to excise themselves from precursor protein without the requirement of any external cofactor or energy. In fact, in a past article, Evans et al. introduced inteins as nature’s escape artists [77]. Intein splicing mechanism requires sequential nucleophilic displacement reactions, similar to serine or cysteine proteases followed by the covalent binding of exteins [3]. Initiation of the splicing pathway requires proper folding of the intein along the N- and C-terminal extein~intein junctions, referred to as splice junctions or sites, so that the nucleophilic residues fall in catalytic range in cases of either contiguous or split-inteins [3,78].

**Figure 5:**
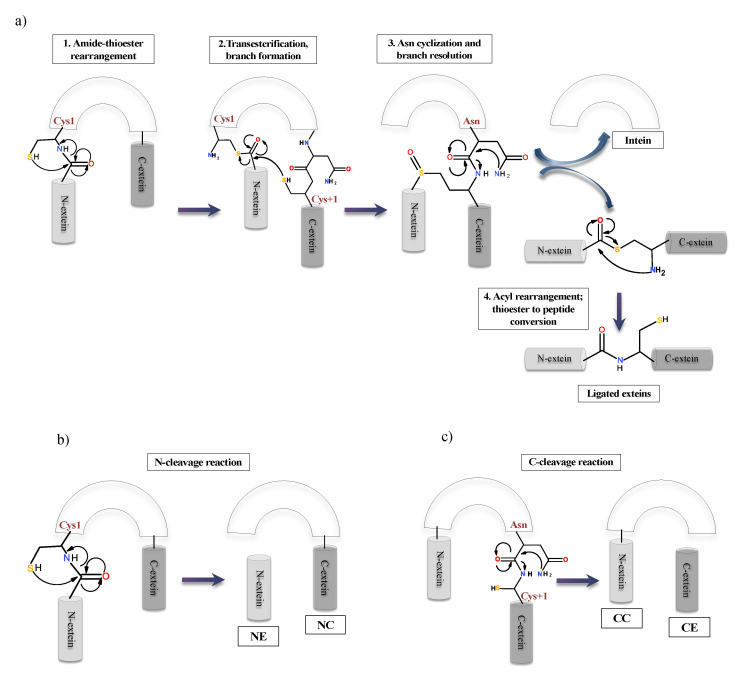
Schematic representation of canonical pathway of intein splicing. (**a**) Intein splicing requires 4 sequential nucleophilic displacement reactions catalysed by Cys1, Cys+1 and terminal Asn. 1. (N-S/N-O) acyl shift converting the peptide bond of N-terminal splice junction to a (thio)ester linkage. 2. A transesterification reaction to form a branched intermediate. 3. Asn cyclization to resolve the branched intermediate by cleavage of C-terminal splice junction. 4. A second (S-N/O-N) acyl shift to ligate the two extein segments by an amide bond formation; (**b**,**c**) off-pathways generating cleavage products. NC: N-terminal cleavage products, CC: C-terminal cleavage products, NE: N-extein and CE: C-extein.

To date, there are three classes of inteins identified with distinct sequence motifs or blocks and splicing strategy. Class 1 or classical (canonical) intein splicing (Figure 5a) involves (1) an (N-S/N-O) acyl shift converting the peptide bond of N-terminal splice junction to a (thio)ester linkage, (2) a transesterification reaction to form a branched intermediate, (3) Asn cyclization to resolve the branched intermediate by cleavage of C-terminal splice junction and (4) a second (S-N/O-N) acyl shift to ligate the two extein segments by an amide bond formation [3,79]. The residues in these blocks are not conserved in entirety, however; several positions on these blocks are highly conserved. Each step is a result of the cumulative action of a few nucleophiles such as Cys1 or Ser1 in step 1; Cys+1, Ser+1 or Thr+1 in step 2 and 4; the C-terminal intein Asn (G-Block) in step 3 and assistance from other known associated residues such as B-Block Thr and His, a penultimate His in G-Block and an F-Block Asp [3,80,81].

The first step of classical or canonical splicing is carried out critically by Block A residues consisting of either Cys, Ser or Thr. Cys, Ser or Thr are essential residues that act as nucleophile in the splicing reaction. This N-O/-S acyl rearrangement is challenging in terms of the kinetic aspect. It needs the assistance from Block B residues: Thr and His, Block F Asp residue and the structural strain in the active site [60,82,83]. The Block B His is a highly conserved residue among all inteins such that any mutations can lead to the generation of inactive precursor or inhibition of cleavage reactions [82,84]. The first N-S acyl shift is catalysed by the Block B His by destabilizing the scissile peptide bond due to a reduction of energy barrier and loss of resonance [83,85]. Both *Sce* VMA and *Mxe* GyrA intein systems show that the Block B His is hydrogen-bonded to the amide nitrogen of the scissile peptide bond. The imidazole ring of His is in proximity for the protonation of the Cys1 amide bond, promoting the breakdown of the linear thioester intermediate yielding an N-extein linked to the intein by a thioester bond. This drives forward the first N-S acyl shift [86,87]. A chemical manipulation at N-splice junction is known to bypass the need of the Block B His for thioesterification by providing the structural strain in *Ssp* DnaB [88]. Moreover, different studies have proposed a dual nature of the Block B histidine, where, as a weak base, it deprotonates the Cys1 to accelerate the N-S acyl shift and as an acid it stabilises the tetrahedral intermediate [89]. A drastic pK_a_ shift in Block B His from a high pK_a_ to a low pK_a_ during the first step of splicing reaction in *Mtu* RecA intein system serves as evidence for its dual role in catalysis [89]. Studies on *Mtu* RecA intein shows that thiol deprotonation is achieved by conserved F-Block Asp and His, causing ground-state destabilization. This destabilization becomes the driving force for the thioesterification step [83,90]. Intein systems lacking the conserved Block B His include *Arthobacter* species FB24 Arth_1007 (DnaB), a degraded pseudogene [91], and *Thermococcus kodakaraensis* Tko CDC21-1 uses a Lys to stabilize the initial N-S acyl shift tetrahedral intermediate, thereby activating N-terminal splice junction [92]. N-exteins residues were also shown to influence N-terminal reactions by Van der Waals forces in *Pyrococcus horikoshii* Pho RadA intein [93]. In another study, +2 C-extein residue in *Nostoc punctiforme* Npu DnaE intein is shown to affect splicing by occupying space at the active site to align the catalytic residues optimally [94]. The F-Block Asp plays a pivotal role in splicing. It is conserved in 60% of intein system and is extensively studied in *Mtu* RecA system [60,90]. Mutational analysis, X-ray crystallographic studies and MD simulations show a flexible conformation by the F-Block, which interacts with both N- and C-splice junction, affecting amide to thioester conversion at both splice sites by acting as a charge relay system [60].

The second step of *trans*-thioesterification is a challenging step to be analysed experimentally since it is tedious to isolate the linear thioester intermediates. This reaction is carried out by the C+1 residue present at C-splice junction. It attacks the linear thioester and forms a branched intermediate structure. The F4 (fourth residue in F block) Asp is linked to this step for deprotonation of C+1, increasing the nucleophilicity of this residue substantially [60]. Deprotonation of C+1 stabilizes the net positive charge on Cys1, helping branch intermediate formation. This is supported by mutational analysis and NMR pK_a_ analysis [90]. The first two steps of splicing are achieved by a complex phenomenon of ground-state destabilization and proton transfer networking among Cys, His, Thr and Asp at A, B and F-Blocks, respectively [60,89,90].

The third step, i.e., Asn cyclization, is irreversible and coupled to the first two steps of splicing reaction. It cleaves the amide bond between the intein and C-extein. Multiple studies have proposed that both F-Block and G-Block histidines are crucial for the coordination of Asn cyclization. The F-Block His helps in increasing the nucleophilicity of terminal Asn by deprotonation and the tetrahedral intermediate stabilization is promoted by both F- and G-Block His. G-Block His increases electrophilicity of the backbone peptide, accelerating the cyclization process [86,95,96,97]. Kinetic studies confirm that the resolution of the branched-chain intermediate to be the rate-limiting step, more so, the Asn cyclization is 200-fold faster with the formation of the branched intermediate [81]. Other studies focus on the local conformations attained by the C-terminal splice site and C-extein after branched intermediate formation.

The fourth and final step involves the conversion of an ester to amide bond between the ligated extein segments. This step does not require any support from either residue of intein or extein, is energetically favourable and faster than the overall reaction [98]. Splicing reaction also generates some off-pathways products as N- or C-cleavage due to mutation at catalytic residues or challenging environmental conditions, which disrupt the normal splicing process (Figure 5b,c). It happens at N- or C-terminal splicing junctions. The (thio)ester bond formed in steps 1 and 2, maybe cleaved due to thiolysis, in a process called N-terminal cleavage. This results in the separation of N-extein from the precursor. Another possibility is the uncoupling of Asn cyclization from steps 1 and 2, liberating the C-extein in a process called C-terminal cleavage [26,79,99].

**Figure 6:**
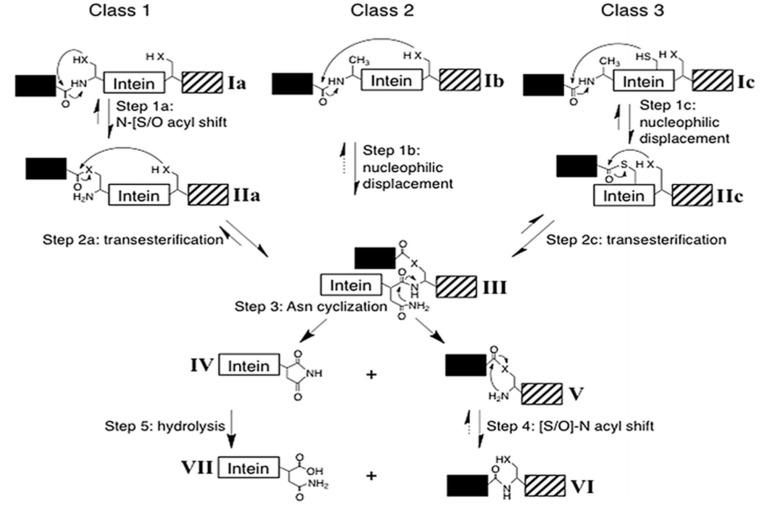
Intein-mediated protein splicing mechanisms in 3 classes of inteins. The majority of inteins follow the class 1 intein-mediated protein splicing mechanism, which consists of four coordinated nucleophilic displacements and requires Ser1, Thr1 or Cys1 as the intein N-terminal residue. Step 1a results in a linear (thio)ester intermediate and step 2a results in BIG with Cys+1, Ser+1 or Thr+1 as the branch point. Class 2 and 3 inteins do not require an intein N-terminal nucleophile. Class 2 inteins directly form BIG when the +1 residue attacks the N-terminal splice junction peptide bond. Class 3 inteins use a conserved Cys at Block F position 4 (CysF:4) to initiate protein splicing resulting in formation of the class-specific BIF. Once BIG is formed, the remaining reactions are the same for all inteins. The acyl shift in step 4 is rapid and spontaneous. Step 5 is also spontaneous but is often slow. Solid arrows represent steps that have been experimentally verified while dashed arrows represent theoretical steps. Note that steps 1 and 2 are reversible; the forward reactions are driven by kinetic rates, equilibrium positions toward the forward reaction, and substrate/intermediate elimination as the protein moves toward the final products, among other factors. Intein residues and flanking extein residues that assist these reactions are not shown, nor are tetrahedral intermediates. X represents the sulphur or oxygen atom in the side chain of Cys, Ser or Thr [104].

Intein containing noncanonical residues implement splicing using variation in the classical pathway (Figure 6). Such inteins fall under class 2 and class 3. Class 2 inteins are also known as Alanine-inteins due to the presence of Ala1 instead of a Cys1 or Ser1. A typical example of class 2 intein is *Mja* KlbA intein, having an Ala1 and G6 Ser instead of Cys1 and G6 His [100]. Class 2 inteins bypass the first step with Cys+1 residue. The Cys+1 attacks the N-terminal amide bond and forms the branched-chain intermediate at G-Block. The pathway progresses in a manner similar to the classical pathway after this step [84,100]. Class 3 splicing mechanism is fascinating and includes the formation of two branched intermediates, a typical example of this class being *Mycobacteriophage bethlehem* DnaB intein [101]. Tori et al. proposed that such inteins initiate splicing reaction by forming a catalytic triad, Trp–Cys–Thr. Position F4 is occupied by Cys instead of Asp, which facilitates N- and C-terminal cleavage reactions. The nucleophilic attack by F4 Cys induces a first F-Block branched intermediate. The second branched intermediate is formed by the nucleophilic attack on N-extein by +1 residue, thereby transferring the N-extein to the C-junction. The final steps of branched intermediate resolution and peptide ligation is similar to that of canonical splicing pathway [84,101,102,103].

Although intein splicing is a spontaneous process and does not require the help of any environmental or molecular signals, recent work has established that intein splicing activity can be regulated by certain molecular triggers like pH, temperature, redox state, salt concentration and even host protein substrate in a process called conditional protein splicing (CPS) [22,23,24,25,26,27]. This regulatory action suggests that inteins may have evolved to be beneficial to the host through post-translational regulation of protein function [26,105,106,107,108,109,110,111]. *Cis*- and *trans*-splicing intein systems can be further modified to perform controlled cleavage and ligation reaction in response to an array of stimuli [23,32]. In recent years, intein-based controllable cleavages have been implemented in processes like protein purification and post-translational modification of recombinant proteins, to name a few [9,32,112]. Techniques like protein *trans*-splicing (PTS) and expressed protein ligation (EPL) have been used extensively in the production of semisynthetic proteins, efficient production of bispecific IgG antibodies and C-terminal modification of recombinant protein bypassing the native peptide thioester chemical synthesis [113,114,115,116].

## 5. Intein Applications

We intend to describe in detail the recent breakthroughs in intein technology in the field of protein expression to purification, post-translational modification and labelling, inteins as a selectable marker, biosensors and in *trans*-gene expression. PTS has been used for the expression of large genes in adult cardiomyocytes by using a split *Npu* DnaE system. Conventional viral vectors failed to manipulate expression of such large genes due to a packaging limitation, now bypassed using PTS technology [117]. Interestingly a hydrogel-based expression system was devised by Ramirez et al., which can be harnessed in the field of tissue engineering, drug delivery and biofabrication [118]. Inteins have also been used in the field of gene therapy to overcome the packaging limitation of Cas9 protein. This was developed by using a split-Cas9 system for recombination and delivery of the repaired template with the Cas9 nuclease activity remaining intact [119].

### 5.1. Protein Purification

**Figure 7:**
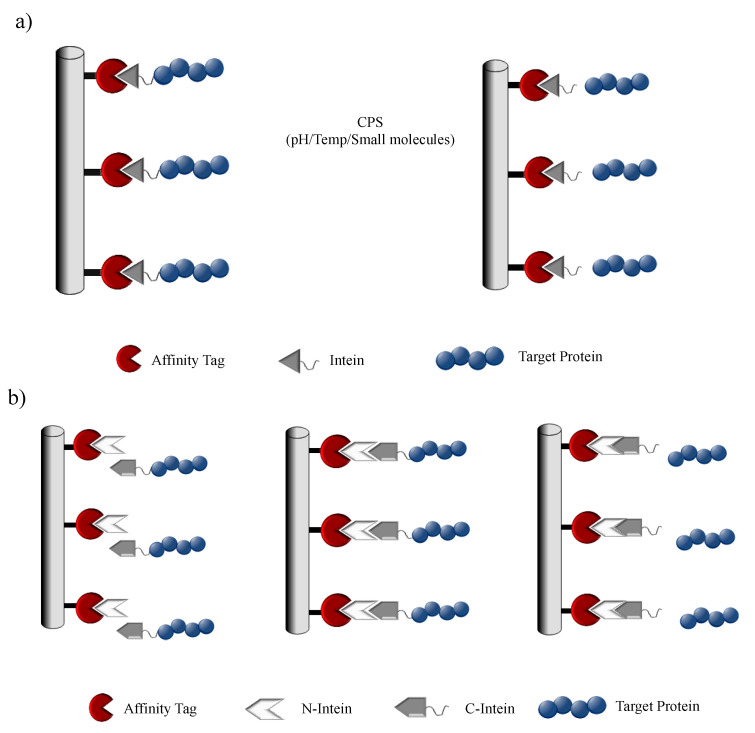
Protein purification system using intein-mediated self-cleaving tag. (**a**) The IMPACT-CN system includes an affinity tag (red) within the intein (grey) and the intein is tagged with a target protein (blue spheres) at C terminal site. (**b**) Schematic representation of split-intein based purification system, where the N-terminal intein is tagged with affinity tag whereas the C-terminal intein is fused with the target protein. The N- and C-intein ligates to form a functional intein segment. Addition of thiol, temperature (Temp) and/or pH changes induce cleavage in the inteins shown above results in isolation of target protein.

With the staggering development in recombinant protein technology, biopharmaceuticals have gained a lot of incentive towards the growth of the pharmaceutical industry. The advances in upstream bioprocessing technology have led to the increased productivities of recombinant proteins in various expression platforms. However, the bottleneck in the production pipeline for recombinant protein expression and modification is evidently the protein purification system. This has led to the development of efficient, rapid and economical downstream unit operations. Conventional downstream processes require multiple steps of product-specific chromatography techniques with long development times, where each step utilizes a particular physical or chemical property of the target. The affinity tag-based purification methods provide a much simpler approach for purifying a broad range of proteins in a highly selective and high-throughput manner. Despite the advantages of the affinity tag-based system, the presence of the tag can pose as an interference with the overall biochemical property of the protein and can be the cause for a potential immunogenic response in the host. Thus, a tag-removal step is highly desirable in the downstream process, but this is usually nonspecific and can denature the target protein or may leave extra amino acids on the target protein post tag removal. Thus, there is a need for applying additional steps to the downstream process, making it more complicated. In the wake of such a complication, intein-based self-cleaving tags were designed to carry out the purification process in a single chromatography step, dramatically reducing the cost and time [120,121,122]. This is achieved by expressing a modified intein fused with an affinity tag along with the target protein. Once the fusion-protein is affinity purified, the intein is induced to cleave the target protein thereby removing the tag along with the intein by harnessing the CPS property. Chong et al. developed a novel protein purification system that reduced purification of free recombinant protein into a single chromatographic step. The system utilized *Sce* VMA intein in conjugation with a chitin-binding domain (CBD) from *Bacillus circulans* as an affinity tag. The target protein to be purified was cloned in frame with this fusion system and under mild conditions induced to undergo self-cleavage as a result of which the target protein is purified while intein–CBD remains bound to the column [120]. In 1997, the first commercial intein system was released by New England Biolabs, which employed a modified *Sce* VMA1 intein, triggered to cleave at its N terminus (IMPACT system) (Figure 7a), or both N and C termini (IMPACT-CN system) by the addition of thiol compounds [32,123]. Similar to the *Sce* VMA intein system, Southworth and coworkers developed a unique and simplified purification system with the help of mini-intein *Mycobacterium xenopi* modified to induce cleavage at a particular temperature or pH [122]. They engineered the mini-intein to generate controllable N-terminal cleavage by adding thiol reagent and C-terminal cleavage product by inducing a temperature shift. The modification of inteins with rapid C-terminal cleaving activity was shortly developed. The establishment of these modified inteins opened avenues for a variety of new tag systems including chitin and maltose-binding protein, nonchromatographic purification tags and small ubiquitin-like modifier (SUMO) to increase expression and purification efficacy [122]. The IMPACT system has been further expanded by New England Biolabs to pTWIN, an analogous system that also uses two CBD-bound inteins [112]. One of the inteins is derived from the split *Ssp* DnaE, modified to perform self-cleavage at its C-terminus. The second intein is derived either from the *Mxe* GyrA gene or from the *Mth* Rir1 gene engineered to perform self-cleavage at their N-termini. Therefore, these dual intein systems allow the stepwise controlled release and purification of target proteins [112].

The most important requisite for all intein-based purification systems is their ability to diminish cleavage during protein expression but cleave rapidly once the fusion precursor is purified. Volkmann and his coworkers demonstrated a new approach to control cleavage by using engineered *Ssp* DnaB split-intein by performing a deletion at the N-terminus, ensuring the eradication of cleaving activity in the intein, allowing the purification of only the C-terminally fused protein target (Figure 7b) [32,124]. The target protein can be obtained by complete removal of the immobilized tag by inducing an intein cleavage by introducing the 11 residue peptide.

Split-inteins offer a possible solution to achieve controllable cleavages without any spontaneous cleavage activity. Such a split-intein-based purification platform was developed by Miguel Ramirez et al. with the help of natural *trans*-splicing *Npu* DnaE intein. It was engineered to exhibit rapid C-terminal cleaving upon reassembly. In this system, the N-terminal segment is fused to an affinity tag and immobilized, while the C-terminal segment is merged to the target protein. By introducing an Asp118Gly single point mutation they were able to modulate the system to undergo accelerated C-terminal cleavage [125]. The mechanism for purification is dependent on the ligation of intein segments, the cleavage reaction suppression by zinc ion followed by rapid target-protein cleavage by thiol incubation [32,125]. A remarkable feature of this intein system is that it could undergo complete cleavage in less than 30 min at room temperature and only in a few hours at 6 °C, thus, making it a potential method for the development of large-scale protein purification techniques.

### 5.2. Protein Modifications

Inteins have been used as a tool to modify the sequence or structure of recombinant protein through reorganization of the peptide bonds. The chemistry of ligating two peptides dates back to the 1990s [20]. These methods include protein cyclization or polymerization, expression of a protein with native N-terminal residue, site-specific labelling and proteolysis [9,32]. The first example of intein-mediated expression system to express, purify and site-specific incorporation of biotin was shown by Lesaicherre et al. in 2002. They expressed fusion proteins with intein tags at C-termini and purified and biotinylated the proteins in a single step to form stable protein microarrays [126].

#### 5.2.1. Protein Backbone Cyclization

**Figure 8:**
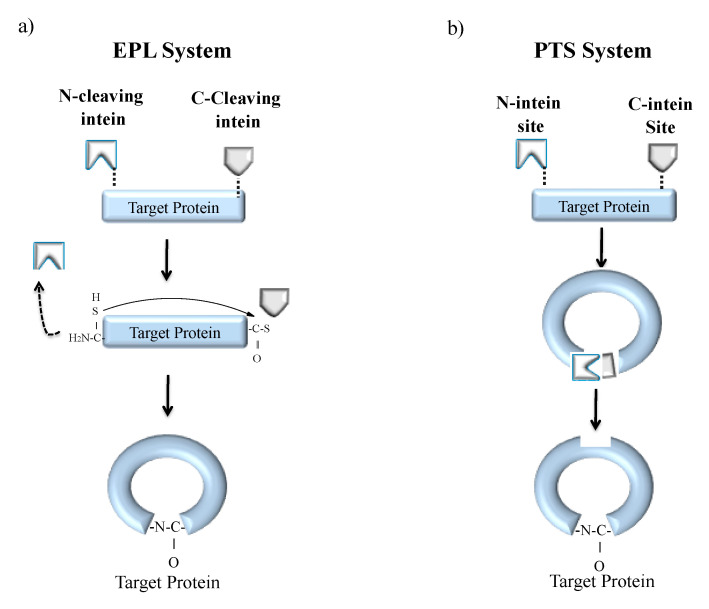
Schematic representation of intein-mediated post-translational modification of a target protein. (**a**) Expressed protein ligation (EPL) systems involve a nucleophilic attack by an N-terminal Cys residue on a thioester formed by a downstream intein. The N-terminal Cys can be generated by a second, upstream intein or by conventional proteolytic cleavage. (**b**) Protein *trans*-splicing (PTS) methods involves cyclization of a target protein tagged with N- and C-intein, which leads to assembly and splicing of an inverted split intein, resulting in generation of a functional target protein.

Cyclization is a process of joining the N- and C-terminal ends of protein via a peptide bond [127]. Naturally cyclized peptides are found in bacteria, plants and mammals that bear antimicrobial, anticancer, uterotonic, haemolytic and anthelmintic property [128,129]. One such naturally occurring cyclized peptide is cyclosporine, found in fungi that harbours an immunosuppressant property [20]. The advantage of polypeptide cyclization is the production of biologically active, fast-folding and denaturation-resistant recombinant proteins. Hence, cyclization of synthetic peptides is an area of interest in pharmaceutical industries. Backbone cyclization can be achieved both in vitro and in vivo systems either by EPL or PTS (Figure 8) [9,32]. The EPL-based cyclization method is achieved by fusing the target polypeptide N-terminus to a peptide leader sequence ending with a Cys residue while the C-terminus is fused to an engineered intein (Figure 8a). The N-terminal leader sequence can undergo cleavage in vivo or in vitro by a proteolytic or self-proteolytic event, leaving behind the N-terminal Cys residue on the target peptide. The Cys residue can then react with an α-thioester generated by the downstream intein, resulting in the formation of a backbone cyclized polypeptide. The first EPL mediated cyclization was reported by Camarero and Muir in 1999 [130]. They used the N-terminal SH3 domain of the c-Crk protein as a model system. The resultant circular protein folded faster and was more stable than the linear counterpart. Iwai and Plückthun used a similar approach for the biosynthesis of a circular beta-lactamase and green fluorescent protein (GFP) that was biologically active and more resistant to thermal denaturation [131].

EPL cyclization technique has also been utilized inside living cells [132]. Camarero and coworkers used Cyclotides (small globular microproteins with a head-to-tail cyclized backbone) and further stabilized it by the formation of a cysteine knot, making them exceptionally resistant to physical, chemical and biological degradation that serves as an ideal scaffold for the development of novel peptide-based therapeutics [133,134].

In a second approach, split inteins were used to produce recombinant cyclized peptides or proteins in vivo, which was also the first reported work by Benkovic and coworkers utilizing PTS (Figure 8b) [135]. The target protein or peptide is expressed as a fusion between C- and N-split intein fragments resulting in enhanced stability and bioactivity [9]. Benkovic and coworkers further exploited PTS technology in combination with nonsense codon suppressor tRNA technology to build libraries of cyclic hexapeptides that include non-natural amino acids. These libraries helped in screening for HIV protease inhibitors using a cell-based lethality assay [136]. PTS technique has also been very successful in the generation of larger circular proteins [135,136,137,138]. For instance, the artificially split *Ssp* DnaB mini-intein has been used by Deschuyteneer et al. to cyclize TEM-1 β–lactamase in the bacterial periplasm, where the split-intein precursor was added to the TEM-1 β–lactamase export signal peptide. This group further produced libraries of circular small peptides using PTS to estimate cyclization efficiency inside living cells. The Kang group also reported that backbone cyclization through PTS can produce intact c-Myc epitope tags for simplification in detection and purification of cyclic products [139].

#### 5.2.2. Protein Processing and Labelling

**Figure 9:**
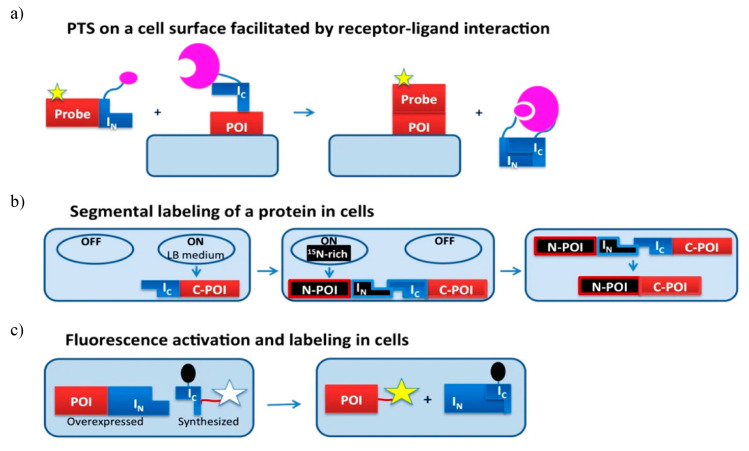
Schematic representations of intein-mediated protein labelling. POI is protein of interest. In (**a**), the complementary pink shapes represent the ligand and its receptor. In (**b**) ”off” and ”on” indicate conditional expression states from a plasmid, with the proteins in black 15 N labelled and those in blue or red are not labelled. In (**c**), the conversion of the star from clear to yellow indicates induction of fluorescence [9].

Protein modification like glycosylation, biotinylation, ubiquitination, phosphorylation, lipidation and segmental isotopic labelling can be done using EPL and PTS technique (Figure 9) [9,32]. Here we discuss intein being harnessed as a tool for different in vivo protein modifications, including protein semisynthesis on cell surfaces, segmental isotope labelling inside the cell and site-specific labelling inside living cells.

PTS technique has been successfully used for protein semisynthesis on cell surfaces. For example, the C-terminus of the human transferrin receptor was labelled with a fluorescent group on the surface of Chinese hamster ovary (CHO) cells using *Ssp* GyrB split intein [140]. Similarly, the N-terminus of the monomeric red fluorescent protein was labelled with biotin on the surface of CHO cell [141]. Mootz and coworker with the help of *Npu* DnaE I_C_ fragment attached enhanced GFP to transmembrane and GPI-anchored proteins [142]. To overcome the low binding affinity between split-inteins constituting short N-terminal fragments, a receptor-ligand interaction was integrated as shown in Figure 9a.

PTS technique can be further implemented for segmental isotopic labelling in vivo as well as the in vivo addition of chemical probes to specific target protein (Figure 9b). Split inteins consisting of either short I_N_ fragment or short I_C_ fragment are used. One such intein-based labelling process uses chemical ligation, to label glutathione-S-transferase (GST) and eGFP in both bacterial and mammalian systems. The target proteins are expressed as a C-terminal fusion to Ssp DnaB intein and an N-terminal Cys. The target protein is tagged using cell-permeable, thioester-containing small molecules like biotin or a fluorophore [143]. However, due to the absence of native affinity between the target protein and label, an excess of one reagent was required for successful ligation at the cost of a high background signal of unreacted reagent. Camerero and coworkers overcame these difficulties and used fluorescence resonance energy transfer (FRET) quenched with DnaE split intein in living cells to increase the affinity between target protein and probe [144]. The fluorescent label was a part of C-extein and the quencher was introduced on the I_C_ intein segment to reduce the background signal. The PTS reaction results in ligation of fluorophores to the protein of interest (POI); subsequently, the quencher is released resulting in fluorescence activity (Figure 9c). A single culture can be used to produce labelled and unlabelled precursor fragments. The need for this dual expression system allows for the sequential expression of precursors in a media spiked with different labelling isotopes. The incorporation of unlabelled tags into isotopically labelled target protein was demonstrated by Züger and Iwai by sequentially overexpressing an unlabelled immunoglobulin binding protein G domain B1 (GB1) and labelled C-terminus of yeast prion protein Sup35, each fused to *Ssp* DnaE split-intein fragment. This fusion protein had improved solubility and stability provided by the NMR-invisible tag [145].

Muir and coworkers used an ultrafast *Npu* split-intein system to incorporate site-specific changes on the chromatin using modified histones via PTS. The I_N_ is fused to the desired histone and the I_C_ fragment is fused to the probe of interest. The reassembly of fragments leads to the generation of semisynthetic histone with the excision of the intein fragment [146]. This group further presented a synthetic approach termed “chemical bait and trap” to assemble engineered histone proteins, using ultrafast *Cfa* split-intein that assists in the incorporation of desired histone post-translational modifications (hPTMs) and cross-linkers. The split inteins present in the truncated histone and the delivery cargo assemble upon PTS, precisely joining the modified histone on the native chromatin [147]. The engineering of reporter proteins was done by Kawase et al. by constructing engineered *Npu* DnaE split intein variants, where the N-intein sequence was modified by inducing Gly4Tyr and Asp5Glu mutations and the C-inteins variants were selected from libraries created by error-prone PCR. Active variants were screened by using a GFP-intein conjugates, which were used to construct a turn-on system for enzymes like human immunodeficiency 1 protease and NanoLuc luciferase [148].

### 5.3. Inteins as a Genetically Selectable Marker

**Figure 10:**
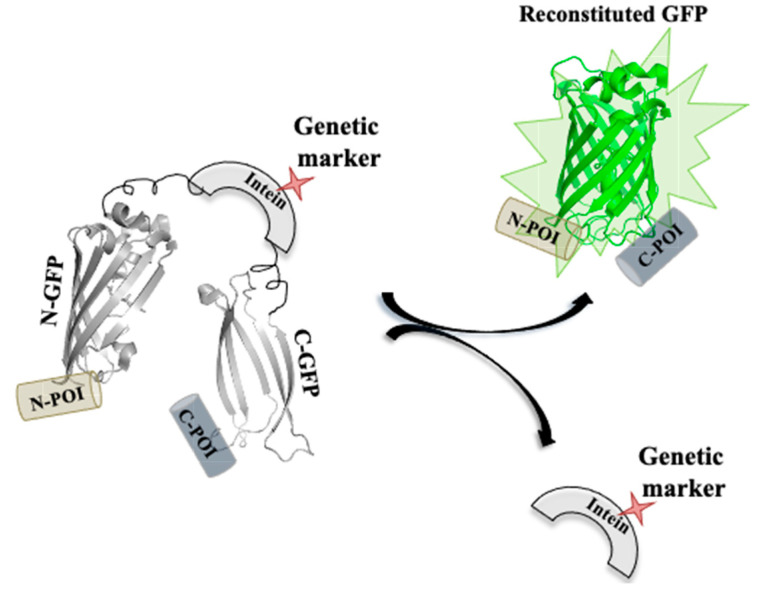
Schematic representation of intein use as a genetically selectable marker. The intein sequence is interrupted by selectable markers. In a suitable genetic background, the intein excises out resulting in the expression of reconstituted green fluorescent protein (GFP) fused with extein fragments on either side. POI is protein of interest.

Inteins can be used as a genetic marker by facilitating in vivo gene modifications (Figure 10). Muller and coworkers modified the *Pch* PRP8 intein system with selectable markers, including amino glycoside, phosphotransferase, imidazoleglycerol-phosphate dehydratase hygromycin B phosphotransferase and transcriptional activator LexA-VP16 [149]. The enzymes as selectable markers were inserted at the site of the lost homing endonuclease domain. The interrupted inteins when expressed in *E. coli* had a higher splicing efficacy; moreover, when the modified inteins were expressed from a plasmid in *S. cerevisiae*, it had splicing efficiency greater than 96%. The sensitivity of internal GFP labelling can be further enhanced by the use of a split GFP–intein construct. Modified intein sequence can be fused with split GFP fragments in-frame with specific peptide (extein) sequences at both the ends. The excised intein under selective conditions can also serve as a selectable marker for the expression of reconstituted GFP fused to exteins on either side [9,149]. 

### 5.4. Intein as a Microbial Drug Target

**Figure 11:**
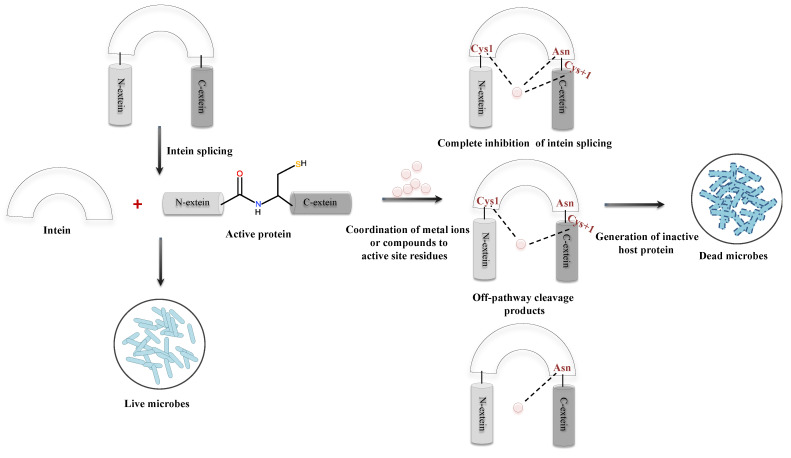
Intein as antimicrobial drug target. Inteins interrupt the functional domains of essential proteins in microorganisms. Removal of inteins generate active proteins, supporting growth and survival of intein-containing microbes. Metal ions or complexes targeting the active site residues can give rise to inactive proteins or off-pathways, splicing byproducts via splicing inhibition, resulting in cell death.

Inteins are sporadically distributed in the genomes of organisms spanning from archaea, bacteria, and eukaryotes to several viral and fungal genomes [20]. Since inteins also intervene in the functional domains of the precursor protein of pathogenic microorganisms, its splicing inhibition can generate an inactive protein that affects microbial viability. Thus, targeting inteins with splicing inhibitors would be a disrupting approach for future antimicrobial development, especially in the era of multidrug resistant (MDR)/extensively drug resistant (XDR) strains [28]. Cisplatin, an anticancer drug, has been extensively used and is a potent inhibitor of splicing mechanism [33]. Nevertheless, cisplatin being an anticancerous drug might possess critical side effects upon administering as an antimicrobial. Transition metal ions such as zinc and copper have also shown substantial ability to inhibit the splicing process. Inhibition of protein splicing is caused by metal coordination to active site residues instead of any structural changes in the protein (Figure 11). Since zinc coordinates to the active site residues, it inhibits protein splicing by restricting the mobility of the active site residues. Copper, however, plays a dual role in the inhibition of intein splicing by strongly coordinating to the key residues (including Cys1) and subsequent oxidation of Cys1 (critical residue for N-S acyl shift, the first step of intein splicing) [150,151]. Metal complexes targeting more than one intein system may have a broad spectrum application as antimicrobials against multiple pathogens. Current studies are conducted either in synthetic intein systems or with engineered mini-intein systems. Studies with native intein systems should thus be acknowledged for development of a novel microbial drug target, particularly for the treatment of infections caused by intein-containing pathogens.

### 5.5. Biosensors

#### 5.5.1. Intein-Based Biosensors

**Figure 12:**
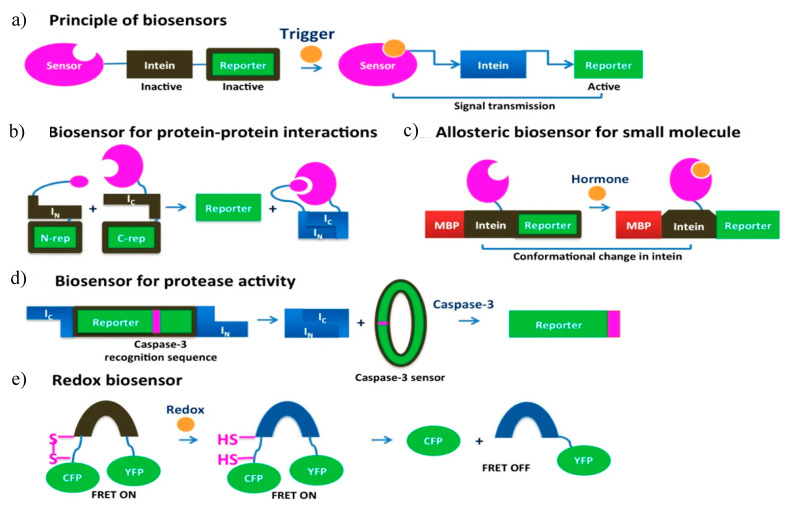
Schematic representation of intein-mediated biosensors. Segments either coloured brown or enclosed in a brown box indicate an inactive intein or reporter. (**a**) Overview of use of an intein as a biosensor. (**b**) Use of an intein-based system to sense protein–protein interactions. (**c**) Intein-mediated allosteric biosensor for small molecules. (**d**) Intein-mediated biosensor for protease activity. (**e**) Intein-mediated redox biosensor. CFP and YFP are cyan and yellow fluorescent proteins, respectively [9].

Intein-based biosensors are fabricated by utilizing independent protein domains, expressed within living cells. Most intein-based biosensor systems have three functional and structural modules: a sensing module, an intein-derived signal transducer, and an output module. CPS has been the foundation that lay to the development of intein-based biosensors. The signal of interest is accepted by the sensing module, which induces CPS in the intein module and subsequent activation of reporter protein, as shown by the output module (Figure 12a). Variation in the development of intein-based biosensors can help detect protein–protein interactions, sensing epigenetic modulations, detection of small molecules, changes is protease activity and redox states, individually. Design strategies with orthogonal split-inteins was used for the development of multiplexed intein-based sensors [152].

#### 5.5.2. Sensing Protein Interactions

Biosensors that detect protein–protein interactions employ the concept of PTS in engineered split-intein systems with low binding affinity between I_N_ and I_C_ fragments. The design criteria for these biosensors includes the formation of two fused proteins, each containing a split-intein and a portion of the reporter molecule. Upon interaction with a binding partner, the split-inteins assemble, resulting in reporter protein reconstitution and activation (Figure 12b). Umezawa et al. used a similar concept to design biosensors to sense protein–protein interaction in in vivo systems ranging from *E. coli* to transgenic animal models. Their original work includes the development of an *E. coli*-based biosensor to monitor the effective binding between calmodulin and M13 (target peptide for calmodulin), using a GFP reconstitution as the reporter, in an artificially split *Sce* VMA1 intein [153]. The group further used similar approaches to detect intracellular interactions between phosphorylated insulin receptor 1 and its target, N-terminal SH2 domain of PI3K [154]. They also demonstrated a noninvasive bioluminescence imaging technique in mice by intein-mediated assembly of split luciferase protein induced by the interaction between MyoD and Id proteins [155]. The sensitivity of detection was enhanced by inducing protein splicing to produce a functional transcription factor that modulates a reporter gene [156]. This work monitors the bioluminescence signals in mammalian cells by monitoring epidermal growth factor (EGF)-induced interaction between oncogenic Ras and its target, Raf-1.

#### 5.5.3. Sensing Epigenetic Modulation

The ability to detect sequence-specific changes in DNA methylation was reported by Huang et al. in living cells. They designed a luminescence-based biosensor for the detection of such epigenetic modulations. The biosensor consists of two fusion proteins, each housing a polydactyl zinc finger domain-split intein fragment-split luciferase domain. Binding of the zinc finger domain to specific DNA target sequences triggers intein-mediated luciferase reporter reassembly [157]. This biosensor design helped in detecting the lack of epigenetic silencing and increased accessibility of a DNA sequence close to the promoter region of L1PA2 subfamily post-treated with demethylation drugs such as 5-azacytidine [9,32].

#### 5.5.4. Sensor for Detecting Small Molecules

Allosteric intein biosensors are used in the detection of small molecules (Figure 12c). Wood et al. designed such a biosensor that detects human nuclear hormone receptor by harnessing the allosteric effects induced by receptor-ligand binding. The sensor, however, does not rely on protein splicing and the intein serves as a medium for signal transduction between the hormone receptor and the reporter. The sensor design includes a four-domain fusion protein with the nuclear receptor of interest present in the loop region of a nonsplicing variant of *Mtu* RecA intein [158]. The intein C-terminus fused to an *E. coli* maltose-binding protein (MBP) and the N-terminus is fused to a T4 bacteriophage thymidylate synthase reporter. The reporter activity is modulated in the presence of hormone in a dose-specific manner, which directly correlates to the growth of *E. coli* cell lines [159]. Past studies used the human estrogen (ERα) and thyroid hormone (TRβ-1) receptors for detecting nuclear hormone receptor ligands [160]. Later, this study led to the development of an optimized estrogen sensor not only capable of identifying diverse estrogen compounds but also distinguishing between agonistic and antagonistic effects [160]. A study by Li et al. utilized the peroxisome proliferator-activated receptor gamma (PPARγ) ligand binding domain to create a series of bacterial biosensors that highlighted the influence on the quality of signal transmission by the thymidylate synthase reporter and the linker region between intein [161].

Buskirk et al. designed a splicing-dependent allosteric intein biosensor for detection of estrogen in an *E. coli* system [23]. The estrogen-sensitive intein was constructed by replacing the endonuclease domain of *Sce* VMA intein with a human ERα receptor ligand binding domain. The DNA coding sequence for this modified intein is then inserted in the chromosomal *lacZ* gene. The sensor follows the principle that the resulting intein will be capable of initiating splicing in the presence of estrogen ligands to produce β-galactosidase reporter enzyme [162].

#### 5.5.5. Redox State Detection

The split *Ssp* DnaE intein system was harnessed for the development of a bacterial redox sensor by utilizing the disulphide-bond control of the intein system (Figure 12e). The sensor was engineered to have a new disulphide bond that includes the N-terminal Cys residue [163]. The disulfide bond trap in the VMA intein is inactive in oxidized form but activates in a reducing environment to generate an N-terminal cleavage. This intein activity is reported by a FRET containing cyan and yellow fluorescent proteins. When the intein remains inactive, the FRET signal is high and upon intein activation, the FRET signal lowers triggering the N-terminal cleavage of the cyan fluorescent protein. This type of redox state biosensors fused with a FRET reporter helps in the detection of hyperoxic *E. coli* mutants [105].

#### 5.5.6. Protease Activity Detection

A biosensor for detecting in-cell protease activity was designed based on intein-mediated protein cyclization (Figure 12d) [164]. A luciferase reporter protein was fused to the caspase-3 recognition sequence, cyclized by the inverted *Ssp* DnaE split intein. In the absence of caspase activity, the cyclized luciferase activity is diminished due to steric hindrance but in presence of caspase-dependent cleavage, the luciferase activity restores. This property helped in the real-time study of caspase-3 presence and activity in live mice.

### 5.6. Transgenic Organisms and Inteins

The combination of split-inteins with PTS has led to the control of *trans*-gene expression [32]. This mechanism relies on the target protein being split into two segments that can later be post-translationally ligated in vivo by PTS [165]. The advantage of this approach is the minimal risk of transfection by particular genes that give rise to desired traits in unwanted host, as in the case of herbicide resistance genes. This technique has been successfully implemented in the split metabolic enzyme acetolactate synthase, reconstituted by PTS in *E. coli* [166]. Similar system design can be seen in *Pseudomonas fluorescens* derived bacterial 5-enolpyruvylshikimate-3-phosphate synthase (EPSPS) reconstitution by PTS in the chloroplasts of both *E. coli* and *Nicotiana tabacum* to produce a herbicide-resistant transgenic crop [167,168]. A split-intein system was also utilized for the generation of transgenic β-glucuronidase (GUS) in *A. thaliana* by PTS and intein-mediated reassembly of GUS fragments [169]. Yang and coworkers extended their previous study in the leaf cells of soybean, maize, barley and pea to show plasmid-induced expression via PTS [170].

Thermostable xylanase (XynB) from *Dictyoglomus thermophilum* is controlled by the thermostable bacterial DnaE-1 intein from *Thermus thermoplilus* in maize [171]. The production of XynB enzyme within the maize plant ensures the production of “self-processing” corn that hydrolyzes its cellulosic biomass to soluble simple sugar for fermentation purposes. The xylanase activity is toxic during the maize growth period since it produces seeds with low seed mass. Thus, the xylanase-intein fusion stops the xylanase from expressing during growth but at high temperatures the XynB-intein construct undergoes splicing and restores the wild-type xylanase activity during biomass production.

PTS has been used recently in mammalian cells and mice to test the delivery and control of large *trans*-genes by adenovirus delivery vectors [172]. The split DnaB mini-intein system has been employed in mammalian cell and in mice for fusion with light and heavy chains of B-domain deleted factor VIII delivered by two separate viral vectors [173]. The in vivo splicing activity was measured by evaluating plasma protein concentration and increased coagulation. These findings suggest that PTS can be used for the in vivo production of an oversized protein too large to be delivered by a single viral vector.

Split-intein system of DnaE has also been used in the production of Cre recombinase in mice [174]. The system is designed such that the fragments of split Cre recombinase is fused to separate promoters that drive expression under different conditions. Under appropriate conditions, when both the Cre fragments are expressed, Cre reconstitution takes place, facilitating the expression of genes under the control of Cre-LoxP system. The split DnaE intein system ensures increased Cre fragment complementation and thus improves its functionality.

### 5.7. Industrial Aspect of Intein Technology

At a research scale, intein-based bioseparations are widely used techniques for single protein purification. The aspect of these purification strategies might eventually lead to advanced application such as large-scale protein production and high-throughput proteomic studies. Albeit, a substantial analysis concerning the economics and feasibility of the potential future systems are required. In principle, conventional affinity tags are used in large-scale manufacturing of purified proteins. However, the economics of the process lies in the affinity tag removal process. At a manufacturing scales of hundred to thousand kilograms per year, these costs rapidly exceed the gross annual sales of even the most lucrative drugs today. Modified inteins can efficiently remove affinity tags by self-cleavage by applying standard conditional splicing techniques (pH and temperature) [32]. The sheer simplicity of this technique suggests that it can be used in large-scale bioseparations. Furthermore, it has been demonstrated to be feasible for proteins expressed under high cell-density conditions, and a self-cleaving tag has also been incorporated successfully into a pilot-scale vortex-flow affinity capture scheme [175,176]. Therefore, the large-scale intein-mediated purification for recombinant proteins has potential economic feasibility.

In a detailed analysis using software simulation of each process (conventional and intein-based), indicated that the operation costs were a major factor for the cost difference, with raw materials accounting for the staggering increase in the cost of intein process. The most expensive raw material was Tris-HCL, which accounted for 61% of all raw materials, followed by DTT, which accounted for 29%. The annual raw material cost-breakdown data reveal the total annual raw material cost for the conventional process was cheaper than the intein-based process. Annual operating expenditure data also reflected that the conventional process is cheaper than the intein-based process. Thus, from this speculation, it is clear that intein-based bioseparations can be economically competitive for large-scale recombinant protein production, but can be more attractive with the use of pH- and temperature-controlled inteins with low-cost buffer systems. The development of more controllable inteins in combination with advanced binding chemistry and process configuration shall increase the opportunity for inteins to be used at a large-scale in the future.

## 6. Conclusions

Inteins are widespread in host proteins and organisms across three kingdoms of life. This extensive phylogenetic distribution pattern of inteins contemplates their nature as a mobile genetic element. Although cases of horizontal intein transfer have been discovered, there remain questions: How does HED associate with inteins? How effortlessly does intein transfer occur among related organisms? Inteins seem to be biased, but not limited to host proteins involved in DNA repair and replication, possibly since it may shuttle intein genes across organisms, help in intein homing and make inteins less harmful to host cell by limiting intein endonuclease production during times of active DNA repair. Laboratory model studies can shed light on the molecular requirements of intein homing. Structure–function studies can also highlight the evolutionary basis of intein invasion and origin, including the structural basis of each step of intein splicing. Inteins can also evolve into new structures harbouring new functions, as seen in cases of *trans*-splicing. The ability to engineer and harness the *trans*-splicing system and other related processes of intein splicing has made inteins very popular in the field of protein engineering, especially in technologies such as purification platforms for biopharmaceutical protein production. Intein research has stepped up from being proof-of-concept experiments to productive application based experiments where inteins are used as tools. They are simple, economic and can be used as either N- or C-terminal cleaving affinity tags, engineered to operate under a wide range of conditions. This resulted in the development of a one-step protein purification strategy for recombinant proteins. However, there are limitations to the intein-engineered purification system. The major limiting factor is premature cleavage during protein expression, followed by product loss during binding capture and the overall need for reducing agents. The introduction of split intein systems has, however, solved the problem of premature cleavage during expression but still requires a reducing agent to induce cleavage. The cleavage activity of inteins controlled by physical conditions suffers from product loss. This shortcoming was partially addressed by the development of nonchromatographic methods such as expressed protein ligation (EPL), although their implementation in large-scale manufacturing will require stringent analysis and optimization to generate better inteins in terms of speed and control of cleavage reactions. Natural and artificially split-intein have not only enhanced conventional technology but also opened new avenues of research in metabolic engineering and drug development. The recent growth in the number of in vivo protein manipulation using intein splicing also guarantees the advancement of intein-based tools. Thus, the future intein technology will build on the present technology to provide new classes of therapeutic proteins and subsequently bridging the gap between systems and functional biology. However, a more detailed characterization of target protein residues at the cleavage junction is required to validate the system to be developed further to be used as a platform. These developing applications as summarised suggest that inteins are becoming more critical and mature biotechnological tools along with the capability of branching into profound areas of research, including the development of newer transgenic plants and novel therapeutic strategies. Despite that critical advances in intein technology have proven to be beneficial in recent years, the economics, scale-up and optimization of intein-mediated techniques at an industrial scale to-date remain a major challenge.
